# Equity or equality in medical education

**DOI:** 10.3352/jeehp.2012.9.3

**Published:** 2012-01-30

**Authors:** Abdul Sattar Khan

**Affiliations:** Family Medicine Department, Ataturk University, Erzurum, Turkey.

Dear editor

I don't fully agree with point of view that Dr. P. Ravi Shankar has mentioned in an article entitled 'Undergraduate medical education in Nepal: one size fits all?'[1]. Of course diversity of students, variations in curriculum, and different teaching approaches may influence the outcomes of health care delivery systems and may lead to inequality in health status. Nevertheless, the curriculum impact assessment is currently under debate, and so far it has not been concluded that one curriculum is fit for all. Therefore, curricula may differ among countries, and even among different contexts in the same country. Certainly the characteristics of different curricula affect performance during medical school and eventually these characteristics are reflected in their practice. This issue is not as straightforward as it appears to be since medical education encompasses different domains.

Contrary to Dr. Shankar's view, I believe that it is already established and evidenced that the core curriculum of basic medical education should be the same for all; however, medical educators should understand that institutions have a responsibility to ensure the production of health personnel who have the knowledge, attitudes, and skills to meet the requirements of their own communities. This educational prerequisite is particularly in need of analysis, and I see this issue in the context of social accountability [2] of health professionals and expect them to understand the health needs of their region, community, and individual patients by interacting with them from the first year to the last year in medical school.

Additionally we cannot ignore "global health," or "international health," a phenomenon that is breaking all boundaries among countries and does not fit into a small cubbyhole. Therefore, I propose that in order to increase the horizon of medical students, medical institutions should be taught a fundamental understanding of the most important diseases that affect and kill people worldwide in order to inform them of the host, environmental, and systems-based factors that govern health worldwide [3].

Importantly, however, medical schools should not need to tailor the whole curriculum for the specific needs of individuals, and I suppose that developing countries like Nepal cannot afford the luxury of offering even a few electives. Hence institutions may include some other domains in the same curriculum, like leadership and management [4], according to local needs in order to understand their own problems in light of factors affecting them from outside of their geographical boundaries.
